# A Stochastic Model for Block Segmentation of Images Based on the Quadtree and the Bayes Code for It †

**DOI:** 10.3390/e23080991

**Published:** 2021-07-30

**Authors:** Yuta Nakahara, Toshiyasu Matsushima

**Affiliations:** 1 Center for Data Science, Waseda University, 1–6–1 Nisniwaseda, Shinjuku-ku, Tokyo 169-8050, Japan; 2 Department of Pure and Applied Mathematics, Waseda University, 3–4–1 Okubo, Shinjuku-ku, Tokyo 169-8555, Japan; toshimat@waseda.jp

**Keywords:** stochastic generative model, quadtree, bayes code, lossless image compression

## Abstract

In information theory, lossless compression of general data is based on an explicit assumption of a stochastic generative model on target data. However, in lossless image compression, researchers have mainly focused on the coding procedure that outputs the coded sequence from the input image, and the assumption of the stochastic generative model is implicit. In these studies, there is a difficulty in discussing the difference between the expected code length and the entropy of the stochastic generative model. We solve this difficulty for a class of images, in which they have non-stationarity among segments. In this paper, we propose a novel stochastic generative model of images by redefining the implicit stochastic generative model in a previous coding procedure. Our model is based on the quadtree so that it effectively represents the variable block size segmentation of images. Then, we construct the Bayes code optimal for the proposed stochastic generative model. It requires the summation of all possible quadtrees weighted by their posterior. In general, its computational cost increases exponentially for the image size. However, we introduce an efficient algorithm to calculate it in the polynomial order of the image size without loss of optimality. As a result, the derived algorithm has a better average coding rate than that of JBIG.

## 1. Introduction

### 1.1. Lossless Data Compression in Information Theory

In information theory, lossless compression for general data (not only images) is based on an explicit assumption of a *stochastic generative model* p(x) on target data x [1]. This assumption determines the theoretical limit, which is called entropy, of the expected code length for p(x). When p(x) is known, entropy codes such as Huffman code [2] and arithmetic code (see, e.g., [3]) achieve the theoretical limit. Then, researchers have considered a setup in which p(x) is unknown. One method to describe the uncertainty of p(x) is removing any specific assumption from the stochastic model p(x) (e.g., [4,5]). Another is considering a class of parameterized stochastic generative models p(x|θ) and assuming the class is known but the parameter θ is unknown. We focus on the latter method in this paper. Even for this setup, researchers have proposed a variety of stochastic generative model classes and coding algorithms achieving those theoretical limits, e.g., i.i.d. model class, Markov model class, context tree model class, and so on (see, e.g., [6,7,8,9,10]).

In this setup, the variety of the stochastic generative model is described as that of unknown parameters or model variables. For example, the i.i.d. model can be determined by a vector θ whose elements are occurrence probabilities of each symbol and described as p(x|θ). Markov model contains another variable *c* that represents the state or context, which is a string of the most recent symbols at each time point, and the occurrence probability vector $\theta_{c}$ is multiplied for each *c*. Then, the Markov model can be described as $p(x|\theta_{c},c)$. Further, when the order of the Markov model is unknown, that contains another variable *k* that represents the order and the occurrence probability $\theta_{c}^{k}$, and the state variables $c^{k}$ are multiplied for each *k*. Then, the Markov model with unknown order can be described as $p(x|\theta_{c}^{k},c^{k},k)$. Moreover, in the context tree model, the order depends on the context, and *k* is replaced by an unknown model variable *m* that represents a set of contexts. Finally, the context tree model can be described as $p(x|\theta_{c}^{m},c^{m},m)$.

It should be noted that these parameters and model variables θ, *k*, and *m* are the *statistical parameters* that govern the generation of the data x. Therefore, the coding algorithm achieving the theoretical limit of these stochastic generative models inevitably contains some kind of statistically optimal action, e.g. their statistical estimation $\hat{\theta }\left(x\right)$, $\hat{k}\left(x\right)$, $\hat{m}\left(x\right)$ as values, their estimation p(θ|x), p(k|x), p(m|x) as posteriors in a Bayesian setting, or model weighting with their posteriors. The explicit assumption of the stochastic generative model and the construction of the coding algorithm with the statistically optimal action have been successful in text compression. In fact, various text coding algorithms have been derived (e.g., [8,9,10]).

### 1.2. Lossless Image Compression as a Image Processing

However, in most cases of lossless “image” compression, the main focus is on the construction of the *coding procedure* f(x) that just outputs the coded sequence from the input pixel values x without the explicit assumption of a stochastic generative model. In the usual case, the coding algorithm has a *tuning parametera* and is represented as f(x;a). This tuning parameter *a* is adaptively tuned to pixel values x, and we express this tuning method as $\tilde{a}\left(x\right)$. Then, the coded sequence $f(x;\tilde{a}\left(x\right))$ from x is uniquely determined.

Therefore, the variety of the coding procedures is described as that of the tuning parameters and the tuning methods. More specifically, we give a brief review of a type of lossless image coding called predictive coding. Most of the predictive coding procedures have a form $f(x^{t-1};a,b)$ with two parameters *a* and *b*. *a* is a parameter of the predictor, which predicts the next pixel value $x_{t}$ from the already compressed pixels $x^{t-1}$ at time *t*. *b* is a parameter that determines an assignment of the code length to the predictive error sequence. Note that the assignment of the code length can be represented by a vector whose sum of the elements equals 1, and it is sometimes called “probability”. However, it does not represent the occurrence probability of pixel value $x_{t}$ in an explicitly assumed stochastic generative model. Therefore, in this paper, we call it *code length assign vector* to distinguish them. Then, the predictive error sequence and the code length assign vector are input to the entropy codes such as the arithmetic code [3]. For example, in JPEG-LS [11], they use three predictors that are switched according to the neighboring pixels. This can be regarded as a∈{1,2,3} corresponding to the index of the three predictors, and the rule to switch them is represented by $\tilde{a}\left(x^{t-1}\right)$. The code length assign vector of JPEG-LS [11] is represented by a two-sided geometric distribution, which is tuned by the past sequence $x^{t-1}$. This can be regarded as *b* being a parameter of the two-sided geometric distribution and $\tilde{b}\left(x^{t-1}\right)$ being its tuning method. In other studies [12,13,14,15,16,17], the authors proposed coding procedures $f(x^{t-1};a,b,c_{a})$ in which coefficients $c_{a}$ of each linear predictor are tuned by a certain method ${\tilde{c}}_{a}\left(x^{t-1}\right)$, e.g., the least squares method or weighted least squares method. In [18,19], the authors proposed coding procedures $f(x^{t-1};a,b,c_{a},w)$ in which multiple predictors are combined according to another tuning parameter w that represents the weights of each predictor. Regarding the code length assign vector, Matsuda et al. [20] dealt with a procedure $f(x^{t-1};a,b,c_{a},d)$ in which the code length assign vector is represented by the generalized Gauss distribution that has another tuning parameter *d*. (This notation is just for the explanation of the idea of the previous studies; it does not completely match the notation of each paper, and it does not contain all of the tuning parameters of each procedure.) One of the latest studies constructing a complicated coding procedure was reported by Ulacha et al. [21], in which numerous tuning parameters are tuned through careful experiments. Lossless image compression using deep learning (see, e.g., [22]) can be regarded as one of the coding procedures with a huge number of tuning parameters that are pre-trained.

These studies have been practically successful. However, it should be noted that the tuning parameters *a* and *b* are not the statistical parameters that govern the generation of pixel values x since they are introduced just to add a degree of freedom to the coding procedure. Even the parameter *b*, which superficially appears to be a parameter of a probability distribution, does not directly govern the generation of pixel values x unless the coding procedure is extremely simple; it is just used to represent the code length assign vector with fewer variables. Therefore, the tuning of these parameters adaptive to x is not theoretically grounded by the statistics nor information theory. If our task were not lossless compression, e.g., lossy compression, image super-resolution, and so on, this parameter tuning would be evaluated from various points of view, e.g., subjective evaluation. It is because such tasks have difficulty in the performance measure itself. Besides, in lossless image compression, it should be evaluated from an information-theoretical perspective. These parameters should be tuned to decrease the difference between the expected code length and the entropy of the assumed stochastic generative model, and we have to say any other tuning methods are heuristic unless they pursue the added value except for the coding rate. However, such an information-theoretical evaluation is impossible because there is no explicit assumption of the stochastic generative model p(x), and the entropy—the theoretical limit of the expected code length—itself is not defined. This is a critical problem of the previous studies above. In addition, the more tuning parameters are introduced, the more difficult the construction of the tuning method becomes since there is no confirmation of the optimality of each tuning method.

### 1.3. Lossless Image Compression on an Explicitly Redefined the Stochastic Generative Model

However, there are some coding procedures f(x;a) [11,12,13,14,16,17,18,19,20,23] whose tuning parameter *a* can be regarded as a statistical parameter of an implicitly assumed statistical generative model p(x|a) by changing the viewpoint. (In some of these studies, the assumption of the stochastic generative model is claimed, but the distinction between the stochastic generative model and the code length assign vector is ambiguous, and the discussion about the difference between the expected code length and the entropy of the stochastic generative model is insufficient.) Further, its parameter tuning method $\tilde{a}\left(x\right)$ could be regarded as a heuristic approximation of a statistically optimal estimation $\hat{a}\left(x\right)\approx \tilde{a}\left(x\right)$. Then, explicitly redefining the implicit stochastic generative model behind the previous coding procedures, we can construct a statistical generative model supported by their practical achievements. Moreover, if we derive the coding algorithm that minimizes the difference between the expected code length and the entropy of the constructed stochastic generative model under some kind of criterion, this algorithm inevitably contains a statistically optimal action that is an improved version of $\tilde{a}\left(x\right)$. Further, such an action is not necessarily the estimation $\hat{a}\left(x\right)$ as a value. We can also estimate its posterior p(a|x) or mix the coding procedures weighted by the posterior p(a|x).

To derive such a coding algorithm, we can utilize the coding algorithms in text coding. Although image data are different from the text data, their stochastic generative models may contain a similar structure, and we may utilize the estimating algorithm in the text coding. In fact, we utilize the efficient algorithm for the context tree model class [8,9,10] for our stochastic generative model in this paper.

It is true that the coding algorithm constructed in this approach does not necessarily work for real images, since the optimality is guaranteed only for the stochastic generative model, and it is difficult to prove that the real images generated from the assumed stochastic generative model. Therefore, the constructed coding algorithm might be inferior to the existing one in the initial stage of this approach. However, we claim that this problem should not be solved by a heuristic tuning of the parameter in the coding procedure but an explicit extension of the stochastic generative model, as much as possible. Such parameter tuning should be done in the final stage before implementation or standardization.

We already adopted this approach in the previous studies [24,25]. In [24], we proposed a two-dimensional autoregressive model and the optimal coding algorithm by interpreting the basic procedure [11,12,13,16,23] of the predictive coding as a stochastic generative model. In [25], we proposed a two-dimensional autoregressive hidden Markov model by interpreting the predictor weighting procedure around a diagonal edge [18] as a stochastic generative model. However, these stochastic generative models do not have enough flexibility to represent the non-stationarity among segments of an image. Therefore, we proposed a stochastic generative model for the non-stationarity in [26]. This paper is an extended version of it.

### 1.4. The Contribution of This Paper

Then, our target data are the images in which the properties of pixel values are different depending on the segments. In this paper, we achieve the following purposes.

1. We propose a stochastic generative model that effectively represents the non-stationarity among the segments in an image.
2. We derive the optimal code that minimizes the difference between the expected code length and the entropy of the proposed stochastic model under the Bayes criterion.
3. We derive an efficient algorithm for the implementation of the code without loss of the optimality.

A trivial way to represent the non-stationarity as a stochastic generative model is to divide the image into fixed-size blocks and assume different probability distributions for each block. However, such a stochastic generative model is not flexible enough to represent the smaller segments and inefficient to represent the larger segments than the block size.

On the other hand, one of the most efficient lossless image coding procedures [20] contains preprocessing to determine a quadtree that represents a variable block size segmentation. Then, different predictors are assigned to each block to mitigate the non-stationarity. The quadtree is also used in various fields of image and video processing to represent the variable block size segmentation, and its flexibility and computational efficiency are reported by a number of studies, e.g., in H.265 [27]. However, the quadtree in these studies is a tuning parameter of a procedure. There are no studies that regard the quadtree as a statistical model variable *m* of a stochastic generative model p(x|m) governing the generation of pixel values x and construct the optimal code that minimizes the difference between the expected code length and the entropy of it in the Bayes criterion, to the best of our knowledge.

In this paper, we propose a novel stochastic generative model based on the quadtree, so that our model effectively represents the non-stationarity among segments by the variable block size segmentation. Then, we construct the optimal code that minimizes the difference between the expected code length and the entropy of the proposed stochastic generative model under the Bayes criterion. The optimal code is given by a weighted sum of all the possible model quadtrees *m*, and the optimal weight is given by its posterior p(m|x). In general, its computational cost increases exponentially for the image size. However, we introduce a computationally efficient algorithm to implement our code without loss of optimality, taking in the knowledge of the text coding [8,9,10]. A similar algorithm is also used for decision tree weighting in machine learning [28]. It is in contrast to the previous lossless image coding procedure [20] that fixes a single quadtree in the preprocessing, which statistically corresponds to some kind of model selection.

Although the main theme of this paper is lossless image compression, the substantial contribution of our results is the construction of the stochastic model. Therefore, the proposed stochastic model contributes to not only lossless image compression but also any other stochastic image processing such as recognition, generation, feature extraction, and so on.

The organization of this paper is as follows. In Section 2, we describe the proposed stochastic generative model. In Section 3, we derive the optimal code for the proposed model. In Section 4, we derive an efficient algorithm to implement the derived code. In Section 5, we perform some experiments to confirm the flexibility of our stochastic generative model and the efficiency of our algorithm. In Section 6, we describe future works. Section 7 is the conclusion of this paper.

## 2. The Proposed Stochastic Model

At first, we define some notations. Note that the following notations are independent of those in Section 1. Let V denote a set of possible values of a pixel. For example, V={0,1} for binary images, V={0,1,⋯,255} for gray scale images, and $V={\left\{0,1,\cdots ,255\right\}}^{3}$ for color images. Let N denote the set of natural numbers. Let h∈N and w∈N denote the height and width of an image, respectively. Although our model is able to represent any rectangular images and its block segmentation, we assume that $h=w=2^{d_{\max}}$ for $d_{\max}\in N$ in the following for the simplicity of the notation. Then, let $V_{t}$ denote the random variable of the *t*th pixel value in order of the raster scan and $v_{t}\in V$ denote its realized value. Note that $V_{t}$ is at x(t)th row and y(t)th column, where *t* divided by *w* is x(t) with a reminder of y(t). In addition, let $V^{t}$ denote the sequence of pixel values $V_{0},V_{1},\cdots ,V_{t}$. Note that all the indices start from zero in this paper.

We consider the pixel value $V_{t}$ is generated from various probability distributions depending on a model m∈M and parameters $\theta^{m}\in \Theta^{m}$. Therefore, they are represented by $p(v_{t}|v^{t-1},\theta^{m},m)$ in general. Note that the model *m* and the parameters $\theta^{m}$ are unobservable and should be estimated in actual situations. The definitions of *m* and $\theta^{m}$ are as follows.

**Definition** **1.** *Let $s_{\left(x_{1}y_{1}\right)\left(x_{2}y_{2}\right)\cdots \left(x_{d}y_{d}\right)}$ denote the following index set called “block”*

(1) $$\begin{array}{cc} s_{\left(x_{1}y_{1}\right)\left(x_{2}y_{2}\right)\cdots \left(x_{d}y_{d}\right)}\left\{\left(i,j\right)\in Z^{2}\;|\;\sum_{d^{\prime }=1}^{d}\frac{x_{d^{\prime }}}{2^{d^{\prime }}}\leq \frac{i}{2^{d_{\max}}}<\left(\sum_{d^{\prime }=1}^{d}\frac{x_{d^{\prime }}}{2^{d^{\prime }}}+\frac{1}{2^{d}}\right),\right.\; & \\ \left.\sum_{d^{\prime }=1}^{d}\frac{y_{d^{\prime }}}{2^{d^{\prime }}}\leq \frac{j}{2^{d_{\max}}}<\left(\sum_{d^{\prime }=1}^{d}\frac{y_{d^{\prime }}}{2^{d^{\prime }}}+\frac{1}{2^{d}}\right)\right\}, \end{array}$$

*where $x_{d^{\prime }},y_{d^{\prime }}\in \left\{0,1\right\}$, $d\leq d_{\max}$, and Z denotes the set of integers. In addition, let $s_{\lambda }$ be the set of whole indices $s_{\lambda }\left\{0,1,\cdots h-1\right\}\times \left\{0,1,\cdots ,w-1\right\}$. Then, let S denote the set which consists of all the above index sets, namely*

(2) $$\begin{array}{c} S\{s_{\lambda },s_{\left(00\right)},\cdots ,s_{\left(11\right)},s_{\left(00)(00\right)},\cdots ,s_{\left(11)(11\right)},\cdots ,s_{(11)(11)\cdots (11}\}. \end{array}$$

**Example** **1.** *For $d_{\max}=2$,*

(3) $$\begin{array}{c} s_{\left(01\right)}=\left\{\left(i,j\right)\in Z^{2}|0\leq i<2,2\leq j<4\right\}=\left\{\left(0,2\right),\left(0,3\right),\left(1,2\right),\left(1,3\right)\right\}. \end{array}$$

*Therefore, it represents the indices of the upper right region. In a similar manner, $s_{\left(01)(11\right)}=\left\{\left(i,j\right)\in Z^{2}|1\leq i<2,3\leq j<4\right\}=\left\{\left(1,3\right)\right\}$. It should be noted that the cardinality |s| for each s∈S represents the number of pixels in the block.*

**Figure 1:**
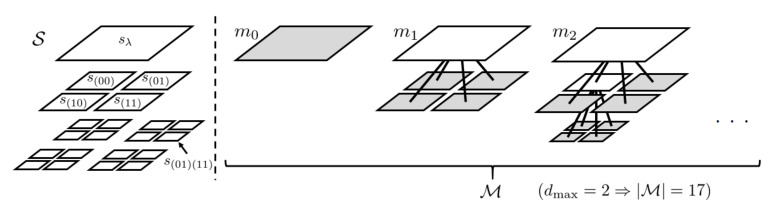
An example of node set S and models *m*.

**Definition** **2.** *We define the model m as a full quadtree whose nodes are elements of S. Let $L^{m}\subset S$ and $I^{m}\subset S$ denote the set of the leaf nodes and the inner nodes of m, respectively. Then, $L^{m}$ corresponds to a pattern of variable block size segmentation, as shown in Figure 1. Let M denote the set of full (i.e., every inner node has exactly four child nodes) quadtrees whose depth is smaller than or equal to $d_{\max}$.*

**Definition** **3.** *Each leaf node $s\in L^{m}$ of the model m has a parameter $\theta_{s}^{m}$ whose parameter space is $\Theta_{s}^{m}$. We define $\theta^{m}$ as a tuple of parameters ${\left\{\theta_{s}^{m}\right\}}_{s\in L^{m}}$, and let $\Theta^{m}$ denote the total parameter space of them.*

Under the model m∈M and the parameters $\theta^{m}\in \Theta^{m}$, we assume that the *t*th pixel value $v_{t}\in V$ is generated as follows.

**Assumption** **1.** *We assume that*

(4) $$\begin{array}{c} p\left(v_{t}|v^{t-1},\theta^{m},m\right)=p\left(v_{t}|v^{t-1},\theta_{s}^{m}\right), \end{array}$$

*where $s\in L^{m}$ satisfies (x(t),y(t))∈s.*

Thus, the pixel value $V_{t}$ depends only on the parameter of the block *s* which contains $V_{t}$ under the past sequence $V^{t-1}$.

## 3. The Bayes Code for the Proposed Model

If we know the true model *m* and the parameters $\theta^{m}$, we are able to compress the pixel value $v_{t}$ up to the entropy of $p(v_{t}|v^{t-1},\theta^{m},m)$ by a well-known entropy code such as the arithmetic code. However, the true *m* and $\theta^{m}$ are unobservable. One reasonable solution is to estimate them and substitute the estimated ones $\hat{m}$ and ${\hat{\theta }}^{m}$ into $p(v_{t}|v^{t-1},\theta^{m},m)$. Then, we can use $p(v_{t}|v^{t-1},{\hat{\theta }}^{m},\hat{m})$ as a coding probability of the entropy code.

However, there is another powerful solution, in which we assume prior distributions p(m) and $p(\theta^{m}|m)$. Then, we estimate the true coding probability $p(v_{t}|v^{t-1},\theta^{m},m)$ itself instead of *m* and $\theta^{m}$ by $q(v_{t}|v^{t-1})$ so that $q(v_{t}|v^{t-1})$ can minimize the *Bayes risk function* based on the *loss function* between the expected code length of entropy code using $p(v_{t}|v^{t-1},\theta^{m},m)$ and that using $q(v_{t}|v^{t-1})$. The code constructed by such a method is called the Bayes code (see, e.g., [29,30]).

It is known that the expected code length of the Bayes code converges to the entropy of the true stochastic model for sufficiently large data length *t*, and its convergence speed achieves the theoretical limits [30]. In fact, the Bayes code achieves remarkable performances in text compression (e.g., [8]).

Therefore, we derive the Bayes code for the proposed stochastic model. According to the general formula in [29], the optimal coding probability for $v_{t}$ in the scheme of the Bayes code is derived as follows:

**Proposition** **1.** The optimal coding probability $q^{*}\left(v_{t}|v^{t-1}\right)$ which minimizes the Bayes risk function is

(5) $$\begin{array}{cc} \; & q^{*}\left(v_{t}|v^{t-1}\right)=p\left(v_{t}|v^{t-1}\right)=\sum_{m\in M}p\left(m|v^{t-1}\right)\int p\left(v_{t}|v^{t-1},\theta^{m},m\right)p\left(\theta^{m}|v^{t-1},m\right)d\theta^{m}. \end{array}$$

We call $q^{*}\left(v_{t}|v^{t-1}\right)$ the Bayes optimal coding probability.

Proposition 1 implies that we should calculate the posterior distributions $p(m|v^{t-1})$ and $p(\theta^{m}|v^{t-1},m)$. Then, we should use the coding probability which is a weighted mixture of $p(v_{t}|v^{t-1},\theta^{m},m)$ for every block segmentation pattern *m* and parameters $\theta^{m}$ according to the posteriors $p(m|v^{t-1})$ and $p(\theta^{m}|v^{t-1},m)$.

## 4. The Efficient Algorithm to Calculate the Coding Probability

Unfortunately, the Bayes optimal coding probability (5) contains computationally difficult calculations. As the depth $d_{\max}$ of full quadtree increases, the amount of calculation for the sum with respect to m∈M increases exponentially. Moreover, the posterior $p(m|v^{t-1})$ does not have a closed-form expression in general. (Strictly speaking, a few problems are also left. Both the integral with respect to $\theta^{m}$ and the posterior $p(\theta^{m}|m,v^{t-1})$ do not have closed-form expressions in general. These problems can be solved in various methods depending on the setting of $p(v_{t}|v^{t-1},\theta^{m},m)$ and $p(\theta^{m}|m)$ and almost independent of our proposed model. Therefore, we describe an example of a feasible setting of $p(v_{t}|v^{t-1},\theta^{m},m)$ and $p(\theta^{m}|m)$ in the next section. Other settings are described in Section 6 as future works.)

Similar problems are studied in text compression, and efficient algorithms to calculate the coding probability have been constructed (see, e.g., [8,9,10]). In these algorithms, the weighted sum of the context trees is calculated instead of the quadtrees. We apply it for our proposed model. In this section, we focus to describe the procedure of the constructed algorithm. Its validity is described in Appendix A.

First, we assume the following priors on *m* and $\theta^{m}$.

**Assumption** **2.** *We assume that each node s∈S has a hyperparameter $g_{s}\in \left[0,1\right]$, and the model prior p(m) is represented by*

(6) $$\begin{array}{c} p\left(m\right)=\prod_{s\in L^{m}}\left(1-g_{s}\right)\prod_{s^{\prime }\in I^{m}}g_{s^{\prime }}, \end{array}$$

*where $g_{s}=0$ for s whose cardinality |s| equals 1, and the empty product equals 1.*

The idea of this form is to represent p(m) as a product of the probability that the block *s* is divided. Such a probability is denoted by $g_{s}$ in (6). Note that |s|=1 means that the block *s* consists of only 1 pixel and it cannot be divided. A proof that the above prior satisfies the condition $\sum_{m\in M}p\left(m\right)=1$ is in Appendix A. Note that the above assumption does not restrict the expressive capability of the general prior in the meaning that each model *m* still has possibility to be assigned a non-zero probability p(m)>0.

**Assumption** **3.** *For each model m∈M, we assume that*

(7) $$\begin{array}{c} p\left(\theta^{m}|m\right)=\prod_{s\in L^{m}}p\left(\theta_{s}^{m}|m\right). \end{array}$$

*Moreover, for any $m,m^{\prime }\in M$, $s\in L^{m}\cap L^{m^{\prime }}$, and $\theta_{s}\in \Theta_{s}$, we assume that*

(8) $$\begin{array}{c} p\left(\theta_{s}|m\right)=p\left(\theta_{s}|m^{\prime }\right)p_{s}\left(\theta_{s}\right). \end{array}$$

Therefore, each element $\theta_{s}^{m}$ of the parameters $\theta^{m}$ depends only on *s* and is independent of both the other elements and the model *m*.

From Assumptions 1 and 3, the following lemma holds.

**Lemma** **1.** *For any $m,m^{\prime }\in M$, $s\in L^{m}\cap L^{m^{\prime }}$, and $v^{t}\in V^{t}$, if (x(t),y(t))∈s, then*

(9) $$\begin{array}{cc} p(v_{t}|v^{t-1},m)\; & =p(v_{t}|v^{t-1},m^{\prime }). \end{array}$$

*Then, we represent it by $\tilde{q}\left(v_{t}|v^{t-1},s\right)$ because it does not depend on m but s.*

The proof of Lemma 1 is in Appendix A. Lemma 1 means that the optimal coding probability for $v_{t}$ depends only on the leaf node block *s* which contains $v_{t}$, and it can be calculated as $q(v_{t}|v^{t-1},s)$ if *s* is known.

At last, the efficient algorithm to compute the Bayes optimal coding probability $q^{*}\left(v_{t}|v^{t-1}\right)$ is represented as an iteration of updating $g_{s}$ and summing the functions $\tilde{q}\left(v_{t}|v^{t-1},s\right)$ weighted by $g_{s}$ for nodes on a path of the complete quadtree on S.

**Figure 2:**
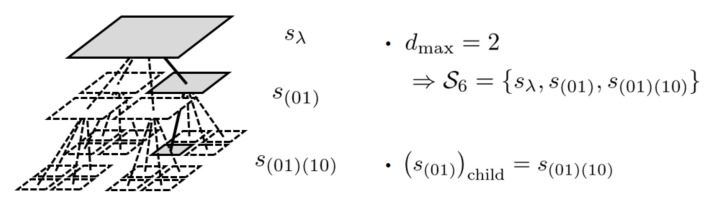
An example of a path constructed from $S_{t}$.

**Definition** **4.** *Let $S_{t}$ denote the set of nodes which contain (x(t),y(t)). They construct a path from the leaf node $s_{\left(x_{1}y_{1}\right)\left(x_{2}y_{2}\right)\cdots \left(x_{d_{\max}}y_{d_{\max}}\right)}=\left\{\left(x\left(t\right),y\left(t\right)\right)\right\}$ to the root node $s_{\lambda }$ on the complete quadtree whose depth is $d_{\max}$ on S, as shown in Figure 2. In addition, let $s_{\mathrm{child}}\in S_{t}$ denote the child node of $s\in S_{t}$ on that path.*

**Definition** **5.** *We define the following recursive function $q(v_{t}|v^{t-1},s)$ for $s\in S_{t}$.*

(10) $$\begin{array}{cc} \; & q\left(v_{t}|v^{t-1},s\right)\left\{\begin{array}{cc} \tilde{q}\left(v_{t}|v^{t-1},s\right), & |s|=1,\\ \left(1-g_{s|t-1}\right)\tilde{q}\left(v_{t}|v^{t-1},s\right)+g_{s|t-1}q\left(v_{t}|v^{t-1},s_{\mathrm{child}}\right), & \mathrm{otherwise}, \end{array}\right. \end{array}$$

*where $g_{s|t}$ is also recursively updated as follows.*

(11) $$\begin{array}{cc} \; & g_{s|t}\left\{\begin{array}{cc} g_{s}, & t=-1\\ g_{s|t-1}, & t\geq 0\wedge (s\notin S_{t}\vee |s|=1)\\ \frac{g_{s|t-1}q\left(v_{t}|v^{t-1},s_{\mathrm{child}}\right)}{q(v_{t}|v^{t-1},s)}, & t\geq 0\wedge s\in S_{t}\wedge \left|s\right|>1. \end{array}\right. \end{array}$$

Then, the following theorem holds.

**Theorem** **1.** The Bayes optimal coding probability $q^{*}\left(v_{t}|v^{t-1}\right)$ for the proposed model is calculated by

(12) $$\begin{array}{c} q^{*}\left(v_{t}|v^{t-1}\right)=q\left(v_{t}|v^{t-1},s_{\lambda }\right). \end{array}$$

The proof of Theorem 1 is in Appendix A. Theorem 1 means that the summation with respect to m∈M in (5) is able to be replaced by the summation with respect to $s\in S_{t}$ and it costs only $O(d_{\max})$. In a sense, $\left(1-g_{s|t-1}\right)$ can be regarded as the marginal posterior probability that the true block division was stopped at *s*. Then, the proposed algorithm takes a mixture of the coding probability $\tilde{q}\left(v_{t}|v^{t-1},s\right)$, weighting such a case with $\left(1-g_{s|t-1}\right)$ and the other cases with $g_{s|t-1}$.

## 5. Experiments

We performed three experiments. The purpose of the first experiment was to confirm the Bayes optimality of $q(v_{t}|v^{t-1},s_{\lambda })$. Therefore, we used synthetic images randomly generated from the proposed model. The purpose of the second experiment was to demonstrate the flexibility of our model. Therefore, we used a well-known benchmark image. We also used the Bayes optimal code for fixed block size segmentation for comparison in these two experiments. (Let $2^{d}$ be the fixed block size. Such a model is derived by substituting $g_{s}=1$ for *s* whose depth is smaller than $d_{\max}-d$ and $g_{s}=0$ otherwise.) The purpose of the third experiment was to compare average coding rates of our proposed algorithm with a current image coding procedure on real images.

### 5.1. Experiment 1

In Experiments 1 and 2, we assumed V={0,1}. In other words, we treated only binary images. $p(v_{t}|v^{t-1},\theta^{m},m)$ was assumed to be the Bernoulli distribution $\mathrm{Bern}(v_{t}|\theta_{s}^{m})$ for *s* which satisfies (x(t),y(t))∈s. Each element of $\theta^{m}$ was i.i.d. distributed with the beta distribution Beta(θ|α,β), which is the conjugate distribution of the Bernoulli distribution. Therefore, the integral in (5) had a closed-form. The hyperparameter $g_{s}$ of the model prior was $g_{s}=1/2$ for every $s\in S\setminus \{s_{\lambda }\}$ and $g_{s_{\lambda }}=1$, and the hyperparameters of the Beta distribution were α=β=1/2.

The setting of Experiment 1 was as follows. The width and height of images were w=h=$2^{d_{\max}}=64$. Then, we generated 1000 images according to the following procedure.

1. Generate *m* according to (6).
2. Generate $\theta_{s}^{m}$ according to $p(\theta_{s}^{m}|m)$ for $s\in L^{m}$.
3. Generate pixel value $v_{t}$ according to $p(v_{t}|v^{t-1},\theta^{m},m)$ for t∈{0,1,⋯,hw−1}.
4. Repeat Steps (1)–(3) 1000 times.

**Figure 3:**
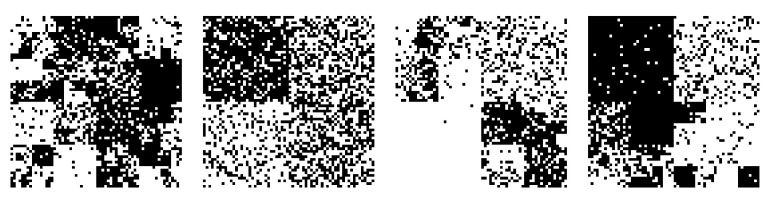
Examples of the generated images in Experiment 1.

Examples of the generated images are shown in Figure 3. Then, we compressed these 1000 images. The size of the image was saved in the header of the compressed file using 4 bytes. The coding probability calculated by the proposed algorithm was quantized in $2^{16}$ levels and substituted into the range coder [31].

The coding rates (bit/pel) averaged over all the images are shown in Table 1. Our proposed code has the minimum coding rate as expected by the Bayes optimality. Additionally, we compressed them by a standard lossless binary image coder called JBIG [32]. It did not work for the generated images. It is probably because JBIG [32] is not designed for synthetic images but mainly for real images such as faxes. A more detailed comparison was done in Experiment 3.

### 5.2. Experiment 2

**Figure 4:**
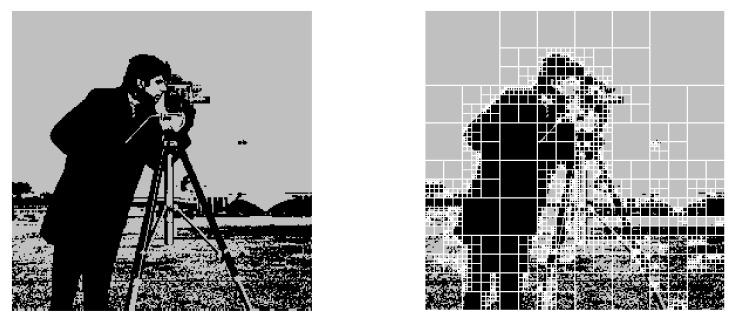
The original image (**left**) and the MAP estimated model $m^{\mathrm{MAP}}$ (**right**).

In Experiment 2, we compressed the binarized version of camera.tif from Wat [33], where the threshold of binarization was 128. The settings of the header and the range coder were the same as those of Experiment 1. Figure 4 visualizes the maximum a posteriori (MAP) estimation $m^{\mathrm{MAP}}=\arg\max_{m}p\left(m|v^{hw-1}\right)$, which was calculated as a by-product of the compression by the algorithm detailed in Appendix B. It shows that our proposed model has the flexibility to represent the non-stationarity among the regions. The coding rate for camera.tif is shown in Table 2. For this image, the proposed algorithm a showed better coding rate than JBIG [32].

### 5.3. Experiment 3

In Experiment 3, we compared the proposed algorithm with JBIG [32] on real images from Wat [33]. They were binarized in a similar manner to Experiment 2. The settings of the header and the range coder were the same as those of Experiments 1 and 2. The results are shown in Table 3. The algorithm labeled as Proposed 1 in Table 3 is the same as that in Experiments 1 and 2. In the algorithm labeled as Proposed 2 in Table 3, we assumed that $p(v_{t}|v^{t-1},\theta^{m},m)$ is the Bernoulli distribution $\mathrm{Bern}(v_{t}|\theta_{s;v_{t-w-1}v_{t-w}v_{t-w+1}v_{t-1}}^{m})$, which depends on the neighboring four pixels. (If the indices go out of the image, we used the nearest past pixel in Manhattan distance.) In other words, there were 16 parameters $\theta_{s;0000}^{m},\theta_{s;0001}^{m},\cdots ,\theta_{s;1111}^{m}$ for each block *s* of model *m*, and one of them was chosen by the realized values $v_{t-w-1}$, $v_{t-w}$, $v_{t-w+1}$, and $v_{t-1}$ in the past. Each parameter was i.i.d. distributed with the beta distribution whose parameters were α=β=1/2.

Proposed 2 outperforms JBIG [32] without any specialized tuning of the hyperparameters from the perspective of average code rates. On the other hand, JBIG [32] outperforms our algorithms for crosses and text. This is because JBIG [32] is designed for text images such as faxes and our stochastic generative model is for images with non-stationarity among segments. The structure of the text images should not be represented by the proposed quadtree-based stochastic generative model but the stochastic model $p(v_{t}|v^{t-1},\theta^{m},m)$ in each block. Although refinement of $p(v_{t}|v^{t-1},\theta^{m},m)$ for target images is out of the scope of this paper, it is an important problem in the future (see the next section).

## 6. Future Works

In this paper, we focus only on the stochastic representation of the non-stationarity among the segments. The discussion about the stochastic model $p(v_{t}|v^{t-1},\theta^{m},m)$ and the prior $p(\theta^{m}|m)$ to be assumed in each block is out of the scope. This is the first future work. For example, our model also works on the pairs of categorical distribution and Dirichlet distribution, normal distribution and normal-gamma distribution, and two-dimensional autoregressive model and normal-gamma distribution [24]. Moreover, using an approximative Bayesian estimation such as the variational Bayesian method, we expect that more complicated stochastic models (e.g., [25]) can be assumed.

The second future work is to apply our model to other stochastic image processing: image recognition, image generation, image inpainting, future extraction, etc. In particular, image generation and image inpainting may be suitable because the whole structure of stochastic image generation is described in our model and the parameters of the stochastic model can be learned optimally.

## 7. Conclusions

We propose a novel stochastic model based on the quadtree so that our model effectively represents the variable block size segmentation of images. Then, we construct a Bayes code for the proposed stochastic model. Moreover, we introduce an efficient algorithm to implement it in polynomial order of data size without loss of optimality. As a result, the derived algorithm has a better average coding rate than that of JBIG [32].

## Figures and Tables

**Table 1:** The average coding rates (bit/pel). The bold number shows the minimum coding rate.

Quadtree (Proposed)	Fixed Size 4	Fixed Size 8	Fixed Size 16	JBIG [32]
**0.619**	0.705	0.659	0.679	1.826

**Table 2:** The coding rates for the camera.tif from Wat [33] (bit/pel). The bold number shows the minimum coding rate.

Quadtree (Proposed)	Fixed Size 4	Fixed Size 8	Fixed Size 16	JBIG [32]
**0.323**	0.427	0.388	0.430	0.348

**Table 3:** The coding rates for the images from Wat [33] (bit/pel). The bold number shows the minimum coding rate.

Images	JBIG [32]	Proposed 1	Proposed 2
bird	0.149	0.121	**0.099**
bridge	0.386	0.390	**0.373**
camera	0.348	0.323	**0.310**
circles	0.102	0.100	**0.060**
crosses	**0.083**	0.140	0.110
goldhill1	0.359	0.371	**0.353**
horiz	0.078	0.075	**0.022**
lena1	0.217	0.254	**0.216**
montage	0.164	0.176	**0.163**
slope	0.096	0.091	**0.056**
squares	0.076	**0.005**	0.010
text	**0.301**	0.468	0.468
avg.	0.197	0.209	**0.187**

**Figure A1:**
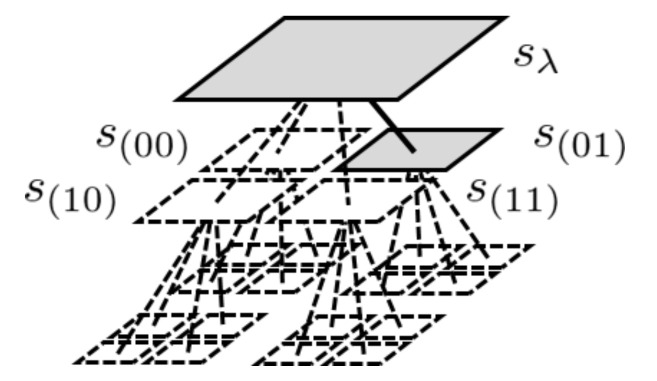
The example for the proof of Corollary A2.

## Data Availability

Publicly available datasets were analyzed in this study. This data can be found here: http://links.uwaterloo.ca/Repository.html (accessed on 30 July 2021).

## Appendix A. Validity of the Proposed Algorithm

### Appendix A.1. The Property of the Model Prior p(m)

First, we prove the following lemma for a general case. Note that the empty product equals 1 as usual.

**Lemma** **A1.**
*Consider the k-ary complete tree $\tilde{T}$ with its depth D, in which each node u has a parameter $g_{u}\in \left[0,1\right]$. Let T denote the set of full subtrees which contain the root node λ of $\tilde{T}$. Then, the following holds.*

(A1) $$\begin{array}{c} \sum_{T\in T}\left(\prod_{u\in L^{T}}\left(1-g_{u}\right)\prod_{u^{\prime }\in I^{T}}g_{u^{\prime }}\right)=1, \end{array}$$

*where $L^{T}$ and $I^{T}$ denote the set of leaf nodes and inner nodes of T, respectively, and $g_{u}=0$ for u whose depth is D.*

**Proof.** Lemma A1 is proved by induction with respect to the depth *D*. Let [λ] denote the tree which consists of only the root node λ of $\tilde{T}$. When D=0,

(A2) $$\begin{array}{cc} \sum_{T\in T}\left(\prod_{u\in L^{T}}\left(1-g_{u}\right)\prod_{u^{\prime }\in I^{T}}g_{u^{\prime }}\right) & =\prod_{u\in L^{\left[\lambda \right]}}\left(1-g_{u}\right)\prod_{u^{\prime }\in I^{\left[\lambda \right]}}g_{u^{\prime }} \end{array}$$

(A3) $$\begin{array}{cc} \; & =1-g_{\lambda } \end{array}$$

(A4) $$\begin{array}{cc} \; & =1, \end{array}$$

where (A2) is because T={[λ]}; (A3) is because $L^{\left[\lambda \right]}=\left\{\lambda \right\}$, $I^{\left[\lambda \right]}=\emptyset$, and the empty product equals to 1; (A4) is because the assumption of the statement, that is $g_{u}=0$ for *u* whose depth is *D*. If we assume (A1) for D=d≥0 as the induction hypothesis, then the following holds for D=d+1.

(A5) $$\begin{array}{cc} \sum_{T\in T}\left(\prod_{u\in L^{T}}\left(1-g_{u}\right)\prod_{u^{\prime }\in I^{T}}g_{u^{\prime }}\right) & =\left(1-g_{\lambda }\right)+\sum_{T\in T\setminus \{[\lambda ]\}}\left(\prod_{u\in L^{T}}\left(1-g_{u}\right)\prod_{u^{\prime }\in I^{T}}g_{u^{\prime }}\right) \end{array}$$

(A6) $$\begin{array}{cc} \; & =\left(1-g_{\lambda }\right)+g_{\lambda }\sum_{T\in T\setminus \{[\lambda ]\}}\left(\prod_{u\in L^{T}}\left(1-g_{u}\right)\prod_{u^{\prime }\in I^{T}\setminus \left\{\lambda \right\}}g_{u^{\prime }}\right). \end{array}$$

Since each subtree T∈T∖{[λ]} is identified by *k* sub-subtrees whose root nodes are the child nodes of λ, let $\lambda_{\mathrm{child},i}$ denote the *i*th child node of λ for 0≤i≤k−1 and $T^{\lambda_{\mathrm{child},i}}$ denote the set of sub-subtrees whose root node is $\lambda_{\mathrm{child},i}$. Then, the summation in (A6) are factorized as follows.

(A7) $$\begin{array}{cc} \; & \sum_{T\in T\setminus \{[\lambda ]\}}\left(\prod_{u\in L^{T}}\left(1-g_{u}\right)\prod_{u^{\prime }\in I^{T}\setminus \left\{\lambda \right\}}g_{u^{\prime }}\right)\\ \; & =\sum_{T_{0}\in T^{\lambda_{\mathrm{child},0}}}\cdots \sum_{T_{k-1}\in T^{\lambda_{\mathrm{child},k-1}}} \end{array}$$

(A8) $$\begin{array}{cc} \; & \;\;\left\{\left(\prod_{u\in L^{T_{0}}}\left(1-g_{u}\right)\prod_{u^{\prime }\in I^{T_{0}}}g_{u^{\prime }}\right)\times \cdots \times \left(\prod_{u\in L^{T_{k-1}}}\left(1-g_{u}\right)\prod_{u^{\prime }\in I^{T_{k-1}}}g_{u^{\prime }}\right)\right\}\\ \; & =\left\{\sum_{T_{0}\in T^{\lambda_{\mathrm{child},0}}}\left(\prod_{u\in L^{T_{0}}}\left(1-g_{u}\right)\prod_{u^{\prime }\in I^{T_{0}}}g_{u^{\prime }}\right)\right\} \end{array}$$

(A9) $$\begin{array}{cc} \; & \;\;\times \cdots \times \left\{\sum_{T_{k-1}\in T^{\lambda_{\mathrm{child},k-1}}}\left(\prod_{u\in L^{T_{k-1}}}\left(1-g_{u}\right)\prod_{u^{\prime }\in I^{T_{k-1}}}g_{u^{\prime }}\right)\right\}. \end{array}$$

Using (A1) for D=d as the induction hypothesis,

(A10) $$\begin{array}{c} \sum_{T_{i}\in T^{\lambda_{\mathrm{child},i}}}\left(\prod_{u\in L^{T_{i}}}\left(1-g_{u}\right)\prod_{u^{\prime }\in I^{T_{i}}}g_{u^{\prime }}\right)=1 \end{array}$$

for 0≤i≤k−1. Then,

(A11) $$\begin{array}{c} \left(A6\right)=\left(1-g_{\lambda }\right)+g_{\lambda }\cdot 1^{k}=1. \end{array}$$

Therefore, Lemma A1 holds for any *D*. □

Using this lemma, the following corollaries hold for our model.

**Corollary** **A1.**
*The prior assumed in Assumption 2 satisfies $\sum_{m\in M}p\left(m\right)=1$.*

**Corollary** **A2.**
*Under Assumption 2 and for any s∈S,*

(A12) $$\begin{array}{c} \sum_{m\in \{m^{\prime }\in M|s\in L^{m^{\prime }}\}}p\left(m\right)=\left(1-g_{s}\right)\prod_{s^{\prime }\in A_{s}}g_{s^{\prime }}, \end{array}$$

*where $A_{s}$ denotes the set of the ancestor nodes of s. (Let $A_{s_{\lambda }}$ be the empty set.)*

**Proof** **of**  **Corollary** **2:** Since each $m\in \{m^{\prime }\in M|s\in L^{m^{\prime }}\}$ has the right-hand side of (A12) as the factor in its prior,

(A13) $$\begin{array}{cc} \; & \sum_{m\in \{m^{\prime }\in M|s\in L^{m^{\prime }}\}}p\left(m\right)\\ \; & \;=\left(1-g_{s}\right)\prod_{s^{\prime }\in A_{s}}g_{s^{\prime }}\sum_{m\in \{m^{\prime }\in M|s\in L^{m^{\prime }}\}}\left(\prod_{s^{\prime }\in L^{m}\setminus \left\{s\right\}}\left(1-g_{s^{\prime }}\right)\prod_{s^{\prime \prime }\in I^{m}\setminus \left\{A_{s}\right\}}g_{s^{\prime \prime }}\right). \end{array}$$

Then, factorizing the sum in a similar manner from (A7)–(A9) and using Lemma A1 for the subtrees whose root nodes are out of $A_{s}$, Corollary A2 is proved. As an example, Figure A1 shows the case where $d_{\max}=2$, $s=s_{\left(01\right)}$, $A_{s_{\left(01\right)}}=\left\{s_{\lambda }\right\}$. Let $M^{s}$ denote a set of full quadtrees whose root node is *s*. In this case, we can factorize the sum in (A13) as follows.

(A14) $$\begin{array}{cc} \; & \sum_{m\in \{m^{\prime }\in M|s_{\left(01\right)}\in L^{m^{\prime }}\}}\left(\prod_{s\in L^{m}\setminus \left\{s_{\left(01\right)}\right\}}\left(1-g_{s}\right)\prod_{s^{\prime }\in I^{m}\setminus \left\{A_{s_{\left(01\right)}}\right\}}g_{s^{\prime }}\right)\\ \; & =\sum_{m_{00}\in M^{s_{\left(00\right)}}}\sum_{m_{10}\in M^{s_{\left(10\right)}}}\sum_{m_{11}\in M^{s_{\left(11\right)}}}\left\{\left(\prod_{s\in L^{m_{00}}}\left(1-g_{s}\right)\prod_{s^{\prime }\in I^{m_{00}}}g_{s^{\prime }}\right)\right. \end{array}$$

(A15) $$\begin{array}{cc} \; & \left.\;\;\;\;\times \left(\prod_{s\in L^{m_{10}}}\left(1-g_{s}\right)\prod_{s^{\prime }\in I^{m_{10}}}g_{s^{\prime }}\right)\left(\prod_{s\in L^{m_{11}}}\left(1-g_{s}\right)\prod_{s^{\prime }\in I^{m_{11}}}g_{s^{\prime }}\right)\right\}\\ \; & =\left\{\sum_{m_{00}\in M^{s_{\left(00\right)}}}\left(\prod_{s\in L^{m_{00}}}\left(1-g_{s}\right)\prod_{s^{\prime }\in I^{m_{00}}}g_{s^{\prime }}\right)\right\}\\ \; & \;\times \left\{\sum_{m_{10}\in M^{s_{\left(10\right)}}}\left(\prod_{s\in L^{m_{10}}}\left(1-g_{s}\right)\prod_{s^{\prime }\in I^{m_{10}}}g_{s^{\prime }}\right)\right\} \end{array}$$

$$\begin{array}{cc} \; & \;\times \left\{\sum_{m_{11}\in M^{s_{\left(11\right)}}}\left(\prod_{s\in L^{m_{11}}}\left(1-g_{s}\right)\prod_{s^{\prime }\in I^{m_{11}}}g_{s^{\prime }}\right)\right\}\\ \; & =\left\{\left(1-g_{s_{\left(00\right)}}\right)+g_{s_{\left(00\right)}}\left(1-g_{s_{\left(00)(00\right)}}\right)\left(1-g_{s_{\left(00)(01\right)}}\right)\left(1-g_{s_{\left(00)(10\right)}}\right)\left(1-g_{s_{\left(00)(11\right)}}\right)\right\}\\ \; & \;\times \left\{\left(1-g_{s_{\left(10\right)}}\right)+g_{s_{\left(10\right)}}\left(1-g_{s_{\left(10)(00\right)}}\right)\left(1-g_{s_{\left(10)(01\right)}}\right)\left(1-g_{s_{\left(10)(10\right)}}\right)\left(1-g_{s_{\left(10)(11\right)}}\right)\right\} \end{array}$$

(A16) $$\begin{array}{cc} \; & \;\times \left\{\left(1-g_{s_{\left(11\right)}}\right)+g_{s_{\left(11\right)}}\left(1-g_{s_{\left(11)(00\right)}}\right)\left(1-g_{s_{\left(11)(01\right)}}\right)\left(1-g_{s_{\left(11)(10\right)}}\right)\left(1-g_{s_{\left(11)(11\right)}}\right)\right\} \end{array}$$

(A17) $$\begin{array}{cc} \; & =1\cdot 1\cdot 1=1. \end{array}$$

The last equation is because $g_{s}=0$ for *s* whose depth is $d_{\max}$. □

### Appendix A.2. Proof of Lemma 1

**Proof** **of** **Lemma** **1.**

(A18) $$\begin{array}{cc} p(v_{t}|v^{t-1},m)\; & =\int p\left(v_{t}|v^{t-1},\theta^{m},m\right)p\left(\theta^{m}|v^{t-1},m\right)d\theta^{m} \end{array}$$

(A19) $$\begin{array}{cc} \; & \propto \int p\left(v_{t}|v^{t-1},\theta^{m},m\right)p\left(v^{t-1}|\theta^{m},m\right)p\left(\theta^{m}|m\right)d\theta^{m} \end{array}$$

(A20) $$\begin{array}{cc} \; & =\int p\left(v_{t}|v^{t-1},\theta_{s}^{m}\right)\int p\left(v^{t-1}|\theta^{m},m\right)p\left(\theta^{m}|m\right)d\theta_{\setminus s}^{m}d\theta_{s}^{m} \end{array}$$

(A21) $$\begin{array}{cc} \; & \propto \int p\left(v_{t}|v^{t-1},\theta_{s}^{m}\right)p_{s}\left(\theta_{s}^{m}\right)\prod_{i\in \{i^{\prime }\leq t|\left(x\left(i^{\prime }\right),y\left(i^{\prime }\right)\right)\in s\}}p\left(v_{i}|v^{i-1},\theta_{s}^{m}\right)d\theta_{s}^{m}, \end{array}$$

where ∝ means that the left-hand side is proportional to the right-hand side, regarding the variables except $v_{t}$ as constant, and $\theta_{\setminus s}^{m}$ denotes the parameters $\theta^{m}$ except $\theta_{s}^{m}$. Here, we use Assumptions 1 and 3. As a result, Formula (A21) is independent of *m*. □

### Appendix A.3. Proof of Theorem 1

**Proof** **of** **Theorem** **1.** We prove the following two equations simultaneously.

(A22) $$\begin{array}{cc} p(m|v^{t-1}) & =\prod_{s\in L^{m}}\left(1-g_{s|t-1}\right)\prod_{s^{\prime }\in I^{m}}g_{s^{\prime }|t-1}, \end{array}$$

(A23) $$\begin{array}{cc} q^{*}\left(v_{t}|v^{t-1}\right) & =q(v_{t}|v^{t-1},s_{\lambda }). \end{array}$$

(A22) means that the posterior distribution of the model *m* has the same form as the prior. (A23) is equivalent to Theorem 1. They are proved by induction with respect to *t*. Therefore, the proof consists of the following four steps.

Step 1 We prove (A22) for t=0.
Step 2 We prove (A23) for t=0.
Step 3 We prove (A22) for t=k+1 under the assumptions of (A22) and (A23) for t=k.
Step 4 We prove (A23) for t=k+1 under the assumptions of (A22) for t=k+1 and (A23) for t=k.

Step 1: (A22) holds for t=0 because it is Assumption 2 itself.
Step 2: For t=0, (A23) can be proved as follows:
  (A24) $$\begin{array}{cc} q^{*}\left(v_{0}\right) & =\sum_{m\in M}p\left(m\right)\int p\left(v_{0}|\theta^{m},m\right)p\left(\theta^{m}|m\right)d\theta^{m} \end{array}$$
  (A25) $$\begin{array}{cc} \; & =\sum_{s\in S_{0}}\sum_{m\in \{m^{\prime }\in M|s\in L^{m^{\prime }}\}}p\left(m\right)\int p\left(v_{0}|\theta^{m},m\right)p\left(\theta^{m}|m\right)d\theta^{m} \end{array}$$
  (A26) $$\begin{array}{cc} \; & =\sum_{s\in S_{0}}\sum_{m\in \{m^{\prime }\in M|s\in L^{m^{\prime }}\}}p\left(m\right)\tilde{q}\left(v_{0}|s\right) \end{array}$$
  (A27) $$\begin{array}{cc} \; & =\sum_{s\in S_{0}}\tilde{q}\left(v_{0}|s\right)\sum_{m\in \{m^{\prime }\in M|s\in L^{m^{\prime }}\}}p\left(m\right) \end{array}$$
  (A28) $$\begin{array}{cc} \; & =\sum_{s\in S_{0}}\tilde{q}\left(v_{0}|s\right)\left(1-g_{s}\right)\prod_{s^{\prime }\in A_{s}}g_{s^{\prime }} \end{array}$$
  (A29) $$\begin{array}{cc} \; & =\left(1-g_{s_{\lambda }}\right)\tilde{q}\left(v_{0}|s_{\lambda }\right)+\sum_{s\in S_{0}\setminus \left\{s_{\lambda }\right\}}\tilde{q}\left(v_{0}|s\right)\left(1-g_{s}\right)\prod_{s^{\prime }\in A_{s}}g_{s^{\prime }} \end{array}$$
  (A30) $$\begin{array}{cc} \; & =\left(1-g_{s_{\lambda }}\right)\tilde{q}\left(v_{0}|s_{\lambda }\right)+g_{s_{\lambda }}\sum_{s\in S_{0}\setminus \left\{s_{\lambda }\right\}}\tilde{q}\left(v_{0}|s\right)\left(1-g_{s}\right)\prod_{s^{\prime }\in A_{s}\setminus \left\{s_{\lambda }\right\}}g_{s^{\prime }}. \end{array}$$

Note that $S_{0}$ is defined in Definition 4. Here, we use Lemma 1 and Corollary A2 in (A26) and (A28), respectively. The recursive structure in (A28) and (A30) coincides with $q(v_{0}|s_{\lambda })$.

Step 3: In the following, we assume (A22) and (A23) for t=k as the induction hypotheses. Let $r\in L^{m}$ satisfy (x(k),y(k))∈r and $S_{k}$ be the same one defined in Definition 4. Then, for t=k+1,
  $$\begin{array}{cc} \; & \prod_{s\in L^{m}}\left(1-g_{s|k}\right)\prod_{s^{\prime }\in I^{m}}g_{s^{\prime }|k} \end{array}$$
  (A31) $$\begin{array}{cc} \; & =\prod_{s\in L^{m}\cap S_{k}}\left(1-g_{s|k}\right)\prod_{s^{\prime }\in I^{m}\cap S_{k}}g_{s^{\prime }|k}\prod_{s^{\prime \prime }\in L^{m}\setminus S_{k}}\left(1-g_{s^{\prime \prime }|k}\right)\prod_{s^{\prime \prime \prime }\in I^{m}\setminus S_{k}}g_{s^{\prime \prime \prime }|k} \end{array}$$
  (A32) $$\begin{array}{cc} \; & =\left(1-g_{r|k}\right)\prod_{s\in A_{r}}g_{s|k}\prod_{s^{\prime }\in L^{m}\setminus S_{k}}\left(1-g_{s^{\prime }|k}\right)\prod_{s^{\prime \prime }\in I^{m}\setminus S_{k}}g_{s^{\prime \prime }|k}. \end{array}$$

When |r|=1, substituting (11) and (10) in this order,

(A33) $$\begin{array}{cc} \left(1-g_{r|k}\right)\prod_{s\in A_{r}}g_{s|k}\; & =\left(1-g_{r|k-1}\right)\prod_{s\in A_{r}}\frac{q(v_{k}|v^{k-1},s_{\mathrm{child}})}{q(v_{k}|v^{k-1},s)}g_{s|k-1} \end{array}$$

(A34) $$\begin{array}{cc} \; & =\frac{\tilde{q}\left(v_{k}|v^{k-1},r\right)}{q(v_{k}|v^{k-1},s_{\lambda })}\left(1-g_{r|k-1}\right)\prod_{s\in A_{r}}g_{s|k-1}. \end{array}$$

Here, (A34) is given by the cancellation of the telescoping product. When |r|>1, substituting (11) and (10) in this order,

$$\begin{array}{cc} \; & \left(1-g_{r|k}\right)\prod_{s\in A_{r}}g_{s|k} \end{array}$$

(A35) $$\begin{array}{cc} \; & =\left(1-\frac{q(v_{k}|v^{k-1},r_{\mathrm{child}})}{q(v_{k}|v^{k-1},r)}g_{r|k-1}\right)\prod_{s\in A_{r}}\frac{q(v_{k}|v^{k-1},s_{\mathrm{child}})}{q(v_{k}|v^{k-1},s)}g_{s|k-1} \end{array}$$

(A36) $$\begin{array}{cc} \; & =\left(\frac{q\left(v_{k}|v^{k-1},r\right)-q\left(v_{k}|v^{k-1},r_{\mathrm{child}}\right)g_{r|k-1}}{q(v_{k}|v^{k-1},r)}\right)\prod_{s\in A_{r}}\frac{q(v_{k}|v^{k-1},s_{\mathrm{child}})}{q(v_{k}|v^{k-1},s)}g_{s|k-1}\\ \; & =\left(\frac{\left(1-g_{r|k-1}\right)\tilde{q}\left(v_{k}|v^{k-1},r\right)+q\left(v_{k}|v^{k-1},r_{\mathrm{child}}\right)g_{r|k-1}-q\left(v_{k}|v^{k-1},r_{\mathrm{child}}\right)g_{r|k-1}}{q(v_{k}|v^{k-1},r)}\right) \end{array}$$

(A37) $$\begin{array}{cc} \; & \;\times \prod_{s\in A_{r}}\frac{q(v_{k}|v^{k-1},s_{\mathrm{child}})}{q(v_{k}|v^{k-1},s)}g_{s|k-1} \end{array}$$

(A38) $$\begin{array}{cc} \; & =\left(\frac{\left(1-g_{r|k-1}\right)\tilde{q}\left(v_{k}|v^{k-1},r\right)}{q(v_{k}|v^{k-1},r)}\right)\prod_{s\in A_{r}}\frac{q(v_{k}|v^{k-1},s_{\mathrm{child}})}{q(v_{k}|v^{k-1},s)}g_{s|k-1} \end{array}$$

(A39) $$\begin{array}{cc} \; & =\frac{\tilde{q}\left(v_{k}|v^{k-1},r\right)}{q(v_{k}|v^{k-1},s_{\lambda })}\left(1-g_{r|k-1}\right)\prod_{s\in A_{r}}g_{s|k-1}. \end{array}$$

Here, (A39) is again given by the cancellation of the telescoping product. As a result, (A34) and (A39) have the same form. On the other hand, applying the updating rule (11),

(A40) $$\begin{array}{c} \prod_{s^{\prime }\in L^{m}\setminus S_{k}}\left(1-g_{s^{\prime }|k}\right)\prod_{s^{\prime \prime }\in I^{m}\setminus S_{k}}g_{s^{\prime \prime }|k}=\prod_{s^{\prime }\in L^{m}\setminus S_{k}}\left(1-g_{s^{\prime }|k-1}\right)\prod_{s^{\prime \prime }\in I^{m}\setminus S_{k}}g_{s^{\prime \prime }|k-1}. \end{array}$$

Therefore, the right-hand side of (A32) is transformed as follows.

(A41) $$\begin{array}{cc} \; & \frac{\tilde{q}\left(v_{k}|v^{k-1},r\right)}{q(v_{k}|v^{k-1},s_{\lambda })}\left(1-g_{r|k-1}\right)\prod_{s\in A_{r}}g_{s|k-1}\prod_{s^{\prime }\in L^{m}\setminus S_{k}}\left(1-g_{s^{\prime }|k-1}\right)\prod_{s^{\prime \prime }\in I^{m}\setminus S_{k}}g_{s^{\prime \prime }|k-1} \end{array}$$

(A42) $$\begin{array}{cc} \; & =\frac{\tilde{q}\left(v_{k}|v^{k-1},r\right)}{q(v_{k}|v^{k-1},s_{\lambda })}\prod_{s\in L^{m}}\left(1-g_{s|k-1}\right)\prod_{s^{\prime }\in I^{m}}g_{s^{\prime }|k-1} \end{array}$$

(A43) $$\begin{array}{cc} \; & =\frac{\tilde{q}\left(v_{k}|v^{k-1},r\right)}{q^{*}\left(v_{k}|v^{k-1}\right)}p\left(m|v^{k-1}\right) \end{array}$$

(A44) $$\begin{array}{cc} \; & =\frac{p(v_{k}|v^{k-1},m)}{p(v_{k}|v^{k-1})}p\left(m|v^{k-1}\right) \end{array}$$

(A45) $$\begin{array}{cc} \; & =p(m|v^{k}). \end{array}$$

In (A43), we use (A22) and (A23) as the induction hypothesis. In (A44), we use Lemma 1 and Proposition 1. Thus, (A22) holds for t=k+1. In addition, it holds that

(A46) $$\begin{array}{c} \sum_{m\in \{m^{\prime }\in M|s\in L^{m^{\prime }}\}}p\left(m|v^{k}\right)=\left(1-g_{s|k}\right)\prod_{s^{\prime }\in A_{s}}g_{s^{\prime }|k}, \end{array}$$

since the posterior $p(m|v^{k})$ has the same form as the prior p(m) and can be applied Corollary A2.

Step 4: (A23) can be proved for t=k+1 in a similar manner to the case where t=0.
  (A47) $$\begin{array}{cc} q^{*}\left(v_{k+1}|v^{k}\right)\; & =\sum_{s\in S_{k+1}}\tilde{q}\left(v_{k+1}|v^{k},s\right)\sum_{m\in \{m^{\prime }\in M|s\in L^{m^{\prime }}\}}p\left(m|v^{k}\right) \end{array}$$
  (A48) $$\begin{array}{cc} \; & =\sum_{s\in S_{k+1}}\tilde{q}\left(v_{t}|v^{k},s\right)\left(1-g_{s|k}\right)\prod_{s^{\prime }\in A_{s}}g_{s^{\prime }|k}\\ \; & =\left(1-g_{s_{\lambda }|k}\right)\tilde{q}\left(v_{k+1}|v^{k},s_{\lambda }\right) \end{array}$$
  (A49) $$\begin{array}{cc} \; & \;+g_{s_{\lambda }|k}\sum_{s\in S_{k+1}\setminus \left\{s_{\lambda }\right\}}\tilde{q}\left(v_{k+1}|v^{k},s\right)\left(1-g_{s|k}\right)\prod_{s^{\prime }\in A_{s}\setminus \left\{s_{\lambda }\right\}}g_{s^{\prime }|k}. \end{array}$$

In (A48), we use (A46). The recursive structure in (A48) and (A49) coincides with $q(v_{k+1}|v^{k},s_{\lambda })$. □

## Appendix B. The Algorithm to Calculate *m*^MAP^

In this appendix, we derive the algorithm to calculate $\arg\max_{m}p\left(m|v^{t}\right)$. At first, $\max_{m}p\left(m|v^{t}\right)$ can be decomposed in a similar manner to the proof of Lemma A1 by replacing the sum for the max.

(A50) $$\begin{array}{cc} \max_{m\in M}p\left(m|v^{t}\right)=\max\{1-g_{s_{\lambda }|t},\; & g_{s_{\lambda }|t}\max_{m_{00}\in M^{s_{\left(00\right)}}}\left\{\prod_{s\in L^{m_{00}}}\left(1-g_{s|t}\right)\prod_{s^{\prime }\in I^{m_{00}}\setminus \left\{s_{\lambda }\right\}}g_{s^{\prime }|t}\right\}\\ \; & \;\times \max_{m_{01}\in M^{s_{\left(01\right)}}}\left\{\prod_{s\in L^{m_{01}}}\left(1-g_{s|t}\right)\prod_{s^{\prime }\in I^{m_{01}}\setminus \left\{s_{\lambda }\right\}}g_{s^{\prime }|t}\right\}\\ \; & \;\times \max_{m_{10}\in M^{s_{\left(10\right)}}}\left\{\prod_{s\in L^{m_{10}}}\left(1-g_{s|t}\right)\prod_{s^{\prime }\in I^{m_{10}}\setminus \left\{s_{\lambda }\right\}}g_{s^{\prime }|t}\right\}\\ \; & \;\times \max_{m_{11}\in M^{s_{\left(11\right)}}}\left\{\prod_{s\in L^{m_{11}}}\left(1-g_{s|t}\right)\prod_{s^{\prime }\in I^{m_{11}}\setminus \left\{s_{\lambda }\right\}}g_{s^{\prime }|t}\right\}\}. \end{array}$$

We define a recursive function $\phi_{t}:S\to R$ as follows.

**Definition** **A1.**

(A51) $$\begin{array}{c} \phi_{t}\left(s\right)\left\{\begin{array}{cc} 1, & |s|=1\\ \max\left\{1-g_{s|t},\;g_{s|t}\phi_{t}\left(s_{{\mathrm{child}}_{00}}\right)\phi_{t}\left(s_{{\mathrm{child}}_{01}}\right)\phi_{t}\left(s_{{\mathrm{child}}_{10}}\right)\phi_{t}\left(s_{{\mathrm{child}}_{11}}\right)\right\}, & \mathrm{otherwise}. \end{array}\right. \end{array}$$

*Here, $s_{{\mathrm{child}}_{00}}$, $s_{{\mathrm{child}}_{01}}$, $s_{{\mathrm{child}}_{10}}$, and $s_{{\mathrm{child}}_{11}}$ are child nodes of s of the complete quadtree on S*

Then, $\max_{m}p\left(m|v^{t}\right)$ can be calculated by $\phi_{t}\left(s_{\lambda }\right).$

Next, we define the following flag variable $h_{s|t}\in \left\{0,1\right\}$.

**Definition** **A2.**

(A52) $$\begin{array}{c} h_{s|t}\left\{\begin{array}{cc} 0, & 1-g_{s|t}\geq g_{s|t}\phi_{t}\left(s_{{\mathrm{child}}_{00}}\right)\phi_{t}\left(s_{{\mathrm{child}}_{01}}\right)\phi_{t}\left(s_{{\mathrm{child}}_{10}}\right)\phi_{t}\left(s_{{\mathrm{child}}_{11}}\right)\\ 1, & \mathrm{otherwise}. \end{array}\right. \end{array}$$

We can calculate $h_{s|t}$ and $\phi_{t}\left(s\right)$ simultaneously. Then, $\arg\max_{m}p\left(m|v^{t}\right)$ is identified as the model which satisfies

(A53) $$\begin{array}{cc} \; & s\in I^{m}\Rightarrow h_{s|t}=1, \end{array}$$

(A54) $$\begin{array}{cc} \; & s\in L^{m}\Rightarrow h_{s|t}=0. \end{array}$$

Such a model can be searched by backtracking from $s_{\lambda }$ after the calculation of $\phi_{t}\left(s_{\lambda }\right)$ and $h_{s_{\lambda }|t}$.
